# Association Between Serum Carnosinase Concentration and Activity and Renal Function Impairment in a Type-2 Diabetes Cohort

**DOI:** 10.3389/fphar.2022.899057

**Published:** 2022-07-08

**Authors:** Jiedong Qiu, Benito A. Yard, Bernhard K. Krämer, Harry van Goor, Peter van Dijk, Aimo Kannt

**Affiliations:** ^1^ 5th Medical Department, University Hospital Mannheim, Heidelberg University, Mannheim, Germany; ^2^ Department of Pathology and Medical Biology, University Medical Centre Groningen and University of Groningen, Groningen, Netherlands; ^3^ Department of Endocrinology, University Medical Centre Groningen and University of Groningen, Groningen, Netherlands; ^4^ Isala, Diabetes Centre, Zwolle, Netherlands; ^5^ Fraunhofer Institute for Translational Medicine and Pharmacology ITMP, Frankfurt, Germany; ^6^ Institute of Experimental Pharmacology, Medical Faculty Mannheim, Heidelberg University, Mannheim, Germany

**Keywords:** diabetic nephropathy, carnosine, histidine-dipeptidase, diabetic complications, carnosinase

## Abstract

**Introduction:** Genetic studies have identified associations of carnosinase 1 (CN1) polymorphisms with diabetic kidney disease (DKD). However, CN1 levels and activities have not been assessed as diagnostic or prognostic markers of DKD in cohorts of patients with type 2 diabetes (T2D).

**Methods:** We established high-throughput, automated CN1 activity and concentration assays using robotic systems. Using these methods, we determined baseline serum CN1 levels and activity in a T2D cohort with 970 patients with no or only mild renal impairment. The patients were followed for a mean of 1.2 years. Baseline serum CN1 concentration and activity were assessed as predictors of renal function impairment and incident albuminuria during follow up.

**Results:** CN1 concentration was significantly associated with age, gender and estimated glomerular filtration rate (eGFR) at baseline. CN1 activity was significantly associated with glycated hemoglobin A1c (HbA1c) and eGFR. Serum CN1 at baseline was associated with eGFR decline and predicted renal function impairment and incident albuminuria during the follow-up.

**Discussion:** Baseline serum CN1 levels were associated with presence and progression of renal function decline in a cohort of T2D patients. Confirmation in larger cohorts with longer follow-up observation periods will be required to fully establish CN1 as a biomarker of DKD.

## Introduction

Carnosinase (CN1) is a dipeptidase, degrading dipeptides with a histidine at the C-terminus such as carnosine, homocarnosine, ophidine, or anserine. These dipeptides are deemed beneficial in diabetes and DKD, as they have pH-buffering, metal-ion chelating, and antioxidative properties as well as the capacity to protect against formation advanced lipid peroxidation end-products (ALEs) and advanced glycation end-products (AGEs) with a higher scavenging capacity towards alpha, beta unsaturated carbonyls compared to reactive dicarbonyls (Boldyrev et al., 2013). The major substrate of CN1, carnosine, consists of beta-alanine and histidine and has been shown to ameliorate hyperglycemia, insulin resistance and albuminuria in animal models (Sauerhöfer et al., 2007; Riedl et al., 2011) and in human (de Courten et al., 2016; Regazzoni et al., 2016). Furthermore, carnosine derivatives such as carnosinol show promising effects in rodent models of carbonyl stress and metabolic disease (Anderson et al., 2018). Thus, elevated CN1 might be deleterious, as it depletes endogenous carnosine levels. Whereas carnosine is the major histidine-containing dipeptide in humans, anserine and ophidine are found in other mammals, and homocarnosine is predominantly found in the central nervous system (Boldyrev et al., 2013).

Although the link between CN1 and DKD has been observed in several studies that found an association of CN1 genetic variants with the risk to develop DKD (Vardarli et al., 2002; Freedman et al., 2007; McDonough et al., 2009; Ahluwalia et al., 2011; Chakkera et al., 2011; Kurashige et al., 2013), CN1 concentration and activity levels have not been measured in large cohorts and assessed as potential diagnostic or prognostic markers.

To assess serum CN1 as a predictor for onset of albuminuria and renal function impairment, we measured CN1 concentration and enzymatic activity in baseline serum samples from a cohort of 970 patients with type 2 diabetes mellitus (T2D) with no or only mild renal impairment.

## Materials and Methods

### Cohort

This is a prospective, observational cohort study. Baseline data and blood samples were obtained from the e-VitaDM study, which was originally designed to assess the feasibility of using an online platform in routine primary healthcare for subjects with T2D (Roelofsen et al., 2014). As a pre-specified part of the e-VitaDM study, patients were assessed over a long-term follow-up period. This prospective arm was nested within the Zwolle Outpatient Diabetes Project Integrating Available Care (ZODIAC) study (Ubink-Veltmaat et al., 2003; Bilo et al., 2009; Landman et al., 2010; Drion et al., 2012; Roelofsen et al., 2014; Alkhalaf et al., 2015; Hendriks et al., 2017). Both the e-VitaDM and the ZODIAC study are described in detail elsewhere (Roelofsen et al., 2014).

This study was conducted in general practices that are connected to the Care Group Drenthe in the Drenthe-region (located in the North-East) of the Netherlands. Fifty-two out of the 110 general practices in this care group agreed to participate. In these practices, approximately 8,300 patients with T2D were treated between 2012 and 2014. Patients were recruited during a regular check-up by their practice nurse and included between May 2012 and September 2014 (Roelofsen et al., 2014; Hendriks et al., 2017). Inclusion criteria were age over 18 years, T2D and the general practitioner as main care provider. Exclusion criteria for e-VitaDM were non-participation in the shared care initiative, mental retardation or psychiatric treatment for schizophrenia, organic mental disorder or bipolar disorder currently or in the past, insufficient knowledge of Dutch language to understand the requirements of the study and/or the questions posed in the questionnaires, life expectancy <1 year due to malignancies or other terminal illnesses, cognitive impairment, including dementia, that interferes with trial participation and any condition that the investigator of coordinating investigator feels would interfere with trial participation or evaluation of results. 1,085 patients (15% of all patients with T2D from the participating general practices) were included. Patients in the Care Group received an annual health check-up. All patients with T2D fulfilling the inclusion criteria, were asked to participate in the prospective observational cohort study (ZODIAC) 3 months before their annual check-up. The study duration lasted 1 year and 3 months and included two annual check-ups. A patient flow-chart can be found in the supplemental materials. For the present study we could obtain serum samples from 970 patients at baseline for determination of CN1 levels and enzymatic activity.

We extracted from the cohort the following parameters which were obtained at the start of the study: age, gender, body mass index (BMI), diabetes duration, glycated hemoglobin (HbA1c), systolic blood pressure (SBP), diastolic blood pressure (DBP), serum creatinine, estimated glomerular filtration rate (eGFR using the Modification of Diet in Renal Disease study formula), urinary albumin creatinine ratio (ACR), presence of microalbuminuria, presence of macroalbuminuria and the use of diabetic and antihypertensive medications. The baseline characteristics are shown in Tables 1, 2. Furthermore we could obtain follow-up data for year one und 2 with a mean follow-up duration of the group of 1.2 years.

**TABLE 1:** Baseline characteristics and CN1 concentration tertiles.

	CN1 Concentration tertiles^b^				
	descriptives^a^	Tertile 1	Tertile 2	Tertile 3	*p* - Value
**Age (years)**	**65.7 (13.4)**	**67.82 (12.3)**	**66.34 (13.1)**	**62.68 (12.4)**	**0.000**
**Gender (% male)**	**54.9%**	**61.8%**	**54.9%**	**47.9%**	**0.002**
CN1 concentration (ng/ml)	1.41 (0.9)	0.80 (90.39)	1.41 (0.30)	2.20 (0.72)	---
BMI (kg/m2)	29.3 (6.2)	29.0 (6.6)	29.3 (5.9)	29.3 (6.3)	0.878
Diabetes duration (years)	6.5 (7.1)	6.5 (6.3)	6.9 (7.0)	5.6 (7.9)	0.098
HbA1c (mmol/mol)	49 (10)	49 (12)	49 (10)	49 (9)	0.905
total cholesterol (mmol/l)	4.3 (1.2)	4.3 (1.2)	4.3 (1.2)	4.3 (1.3)	0.677
HDL cholesterol (mmol/l)	1.2 (0.5)	1.2 (0.4)	1.2 (0.4)	1.2 (0.5)	0.576
LDL cholesterol (mmol/l)	2.3 (1.0)	2.2 (0.9)	2.3 (1.0)	2.4 (1.0)	0.336
triglycerides (mmol/l)	1.5 (1.0)	1.6 (1.2)	1.4 (1.0)	1.5 (1.0)	0.066
SBP (mmHg)	135 (19)	135 (16)	135 (16)	135 (18.75)	0.516
DBP (mmHg)	80 (12)	78 (12)	80 (14)	80 (11)	0.557
**serum creatinine (µmol/l)**	**78 (22.25)**	**81 (23.50)**	**79 (23)**	**75 (21)**	**0.001**
**eGFR MDRD (ml/min/1.73m2)**	**73 (925)**	**71 (25)**	**73 (23.50)**	**76 (24.50)**	**0.001**
Urinary ACR (mg/mmol)	0.80 (1.10)	0.90 (1.10)	0.80 (1.13)	0.70 (1.19)	0.145
Medication use					
Metformin (%)	74.6%	74.4%	77.7%	71.8%	0.240
Sulfonylurea (%)	27.5%	23.9%	30.2%	28.5%	0.202
Thiazolinedione (%)	1.0%	1.0	1.0	1.0	1.000
DDP4 inhibitor (%)	3.8%	2.9%	5.9%	2.6%	0.058
ACE-inhibitor/ARB-blocker (%)	59.1%	57.6%	61.6%	58.3%	0.590
Beta-blocker (%)	40.3%	42.1%	43.4%	35.3%	0.110
Calcium-Antagonist (%)	19.1%	21.4%	18.5%	17.3%	0.444
Diuretics (%)	40.4%	39.0%	45.6%	36.7%	0.086

a Descriptives as median (*IQR*) or frequency (%).

b Tertiles as median (*IQR*) or frequency (%).

Abbreviations: CN1 carnosinase 1, BMI, body mass index, HbA1c glycated hemoglobin, HDL, high density lipoprotein; LDL, low density lipoprotein; SBP, systolic blood pressure; DBP, diastolic blood pressure; eGFR, estimated glomerular filtration rate; ACR, albumin creatinine ratio, DDP4 dipeptidylpeptidase 4, ACE, angiotensin converting enzyme; ARB, angiotensin II, receptor blocker.

Parameters with significant differences between tertiles (*p* < 0.05) are shown in bold print.

**TABLE 2:** Baseline characteristics and CN1 activity tertiles.

	CN1 activity tertiles^b^				
	descriptives^a^	tertile 1	tertile 2	tertile 3	*p* - value
age (years)	65.7 (13.4)	67.6 (15.1)	65.7 (12.5)	65.2 (12.5)	0.094
gender (% male)	54.9%	55.5%	55.7%	53.4%	0.808
CN1 activity (µmol/min/mg)	0.06 (0.05)	0.03 (0.01)	0.06 (0.01)	0.10 (0.03)	---
BMI (kg/m^2^)	29.3 (6.2)	28.9 (6.5)	29.3 (6.0)	29.4 (6.1)	0.810
diabetes duration (years)	6.5 (7.1)	6.5 (7.1)	6.5 (7.2)	6.3 (7.3)	0.645
**HbA1c (mmol/mol)**	**49** **(10)**	**50 (10)**	**49 (10)**	**48 (10)**	**0.012**
**total cholesterol (mmol/l)**	**4.3** **(1.2)**	**4.2 (1.3)**	**4.2 (1.1)**	**4.4 (1.2)**	**0.034**
**HDL cholesterol (mmol/l)**	**1.2** **(0.5)**	**1.2 (0.5)**	**1.2 (0.4)**	**1.3 (0.4)**	**0.007**
LDL cholesterol (mmol/l)	2.3 (1.0)	2.2 (1.1)	2.3 (1.0)	2.4 (1.0)	0.101
triglycerides (mmol/l)	1.5 (1.0)	1.5 (1.0)	1.5 (1.0)	1.6 (1.1)	0.239
SBP (mmHg)	135 (19)	135 (18)	134 (19)	136 (18.25)	0.531
DBP (mmHg)	80 (12)	80 (12)	80 (12)	79 (12)	0.724
serum creatinine (µmol/l)	78 (22.25)	78 (23)	79 (24)	77 (22)	0.124
**eGFR MDRD (ml/min/1.73m^2^)**	**73** **(25)**	**69 (24)**	**73 (26)**	**77 (23)**	**0.000**
urinary ACR (mg/mmol)	0.80 (1.10)	0.70 (1.10)	0.90 (1.10)	0.90 (1.19)	0.463
use of					
Metformin (%)	74.6%	72.8%	74.8%	76.2%	0.621
Sulfonylurea (%)	27.5%	27.5%	26.5%	28.6%	0.837
Thiazolinediones (%)	1.0%	1.0%	1.6%	0.3%	0.252
DDP4 inhibitor (%)	3.8%	4.5%	2.0%	4.8%	0.123
ACE-inhibitor/ARB-blocker (%)	59.1%	56.1%	62.9%	58.5%	0.240
Beta-blocker	40.3%	40.5%	40.6%	39.7%	0.971
**Calcium-Antagonist (%)**	**19.1** **%**	**15.9%**	**23.7%**	**17.8%**	**0.047**
Diuretics (%)	40.4%	39.1%	42.1%	40.1%	0.762

a Descriptives as median (*IQR*) or frequency (%).

b Tertiles as median (*IQR*) or frequency (%).

Abbreviations: CN1 carnosinase 1, BMI, body mass index, HbA1c glycated hemoglobin, HDL, high density lipoprotein; LDL, low density lipoprotein; SBP, systolic blood pressure; DBP, diastolic blood pressure; eGFR, estimated glomerular filtration rate; ACR, albumin creatinine ratio, DDP4 dipeptidylpeptidase 4, ACE, angiotensin converting enzyme; ARB, angiotensin II, receptor blocker.

Parameters with significant differences between tertiles (*p* < 0.05) are shown in bold print.

### Carnosinase 1 Activity and Concentration Measurements

CN1 activity was measured in a total of 970 serum samples. Determination of enzymatic activity was based on the high-throughput method previously described (Qiu et al., 2019). CN1 concentration was measured using the commercial ELISA kit CSB-EL005639HU from Cusabio Technology (United States) according to manufacturer’s instructions. Outliers per standard definition as values above 1.5 times interquartile range from the first and third quartile were eliminated listwise from all analyses (11 patients were excluded due to outliers in the CN1 concentration). From the remaining 959 patients, we could obtain clinical data from 926 patients for the analysis. The CN1 concentration and activities were divided into tertiles and the baseline characteristics were compared across the tertiles.

### Statistics

SPSS Statistics 26 (Windows version, IBM, United States) was used for all statistical analyses. A two-sided alpha level of 0.05 was considered significant.

Baseline characteristics: All variables were checked for normality and outliers before analysis. Outliers were defined as > 1.5 interquartile range (IQR) from he 25th or 75th per-centile and identified on a box plot. Continuous parameters were shown as median (IQR). Nominal data were shown as percentage. Since CN1 concentration and activity were non-normally distributed, Spearman’s rank order correlation coefficient was calculated to assess the relationship between CN1 concentration and activity. The study population was divided into tertiles according to CN1 concentration and activity. All continuous clinical parameters if separated in tertiles by CN1 concentration or activity failed the assumption of normality in every subgroup for ANOVA analysis as tested with the Shapiro-Wilk test. Furthermore, HbA1c also violated the assumption of homogeneity of variance for CN1 concentration subgroups and age for CN1 activity subgroups (Levene statistic *p* < 0.01). Thus, the nonparametric Kruskal–Wallis test was used to analyze different CN1 concentration or activity tertiles on continuous parameters. Chi-square test was used to compare dichotomous data according to CN1 concentration and activity.

The cohort was followed for a mean of 1.2 years. During the follow-up, onset of renal function impairment defined as eGFR below 60 ml/min/1.73 m^2^ and albuminuria defined as microalbuminuria or macroalbuminuria were recorded as dichotomous data. These parameters were used in a multivariable binary logistic regression analysis (method: forced-entry) to study its relationship to the previously extracted clinical data and the CN1 parameters measured at baseline of the study.

We calculated the eGFR decline of patients who were followed for at least 1 year using the eGFR MDRD values. A cutoff of 3 ml/min/1.73 m^2^ per year was used to distinguish those patients with progressive eGFR decline from those who had a no or moderate eGFR decline (eGFR between -3 and +3 ml/min/1.73 m^2^). We then assessed the CN1 concentration and activity levels in both groups using a nonparametric Mann-Whitney *U* test. Missing data were excluded from the analysis pair-wise for each analysis.

### Ethics Approval

The study protocol (study ID METC 11.10117) was approved by the Medical Ethics Committee of the Isala hospital (Zwolle, the Netherlands). The protocol of the clinical trial was registered prior on clinicaltrials.gov (study ID NCT01570140). Informed consent was obtained from each individual, and all procedures were performed in accordance with the Helsinki Declaration.

## Results

### Carnosinase 1 Concentration and Activity

Baseline characteristics are shown in Tables 1, 2. A significant positive association between CN1 activity and concentration was found (ρ 959) = 0.143, *p* < 0.001). The relationship between serum CN1 activity and concentration is shown in Supplementary Figure S1.

### Baseline Characteristics and their Association With Carnosinase 1

**FIGURE 1:**
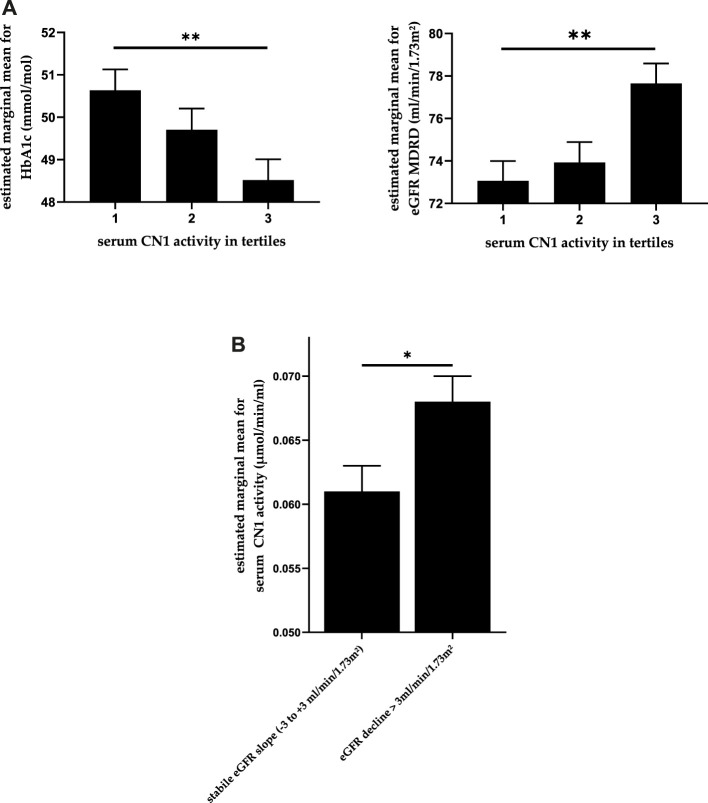
**(A)** Association between CN1 activity and both HbA1c and eGFR whilst controlling for age and gender. The estimated means are depicted in the picture with standard error. **(B)** CN1 activity in patients with an eGFR decline >3 ml/min/1.73 m^2^ per anno compared to those with stable eGFR whilst controlling for age and gender. One-way ANCOVA, *p*-values adjusted using Bonferroni method. * for *p* < 0.05 and ** for *p* < 0.01.

As presented in Tables 1, 2, significant differences in age, baseline serum creatinine and eGFR were found between different CN1 concentration tertiles. Similarly, significant differences in eGFR were also found between CN1 activity tertiles. Furthermore, CN1 activity was also significantly associated with baseline HbA1c whereas CN1 concentration was not. After adjustment for gender and age, there was no longer a significant association of CN1 concentration with eGFR (*p* = 0.151). In contrast, the association of CN1 activity tertiles with HbA1c and eGFR remained statistically significant (*p* = 0.006 and *p* = 0.003 respectively; Figure 1). Furthermore, while there was no significant association of any medications with CN1 concentration, the use of calcium-antagonists seemed to be higher in the middle tertile of CN1 activity.

### Carnosinase 1 and Prediction of Renal Function Impairment

The cohort was followed for a mean period of 1.2 years. The development of clinical characteristics over the follow-up period is summarized in Supplementary Tables S1–S5. We selected patients who had a normal or only mildly decreased eGFR (>60 ml/min/1.73 m^2^) at baseline. Of these 468 patients, 44 (9.4%) showed renal function decline (eGFR below 60 ml/min/1.73 m³) during the follow-up period. A logistic regression analysis showed that there was a significant influence of gender, age, BMI, and baseline CN1 concentration on the worsening of eGFR (Χ^2^ for the model 12) = 34.482, *p* = 0.001). The results showed that for every unit (ng/ml) increase in CN1 concentration the odds ratio to have an eGFR below 60 ml/min/1.73 m^2^ during the follow-up was 0.492 (95% CI: 0.290–0.837, *p* = 0.009). CN1 activity was not associated with worsening of eGFR (*p* = 0.539).

### Prediction of Incident Micro-/Macroalbuminuria During Follow up

**FIGURE 2:**
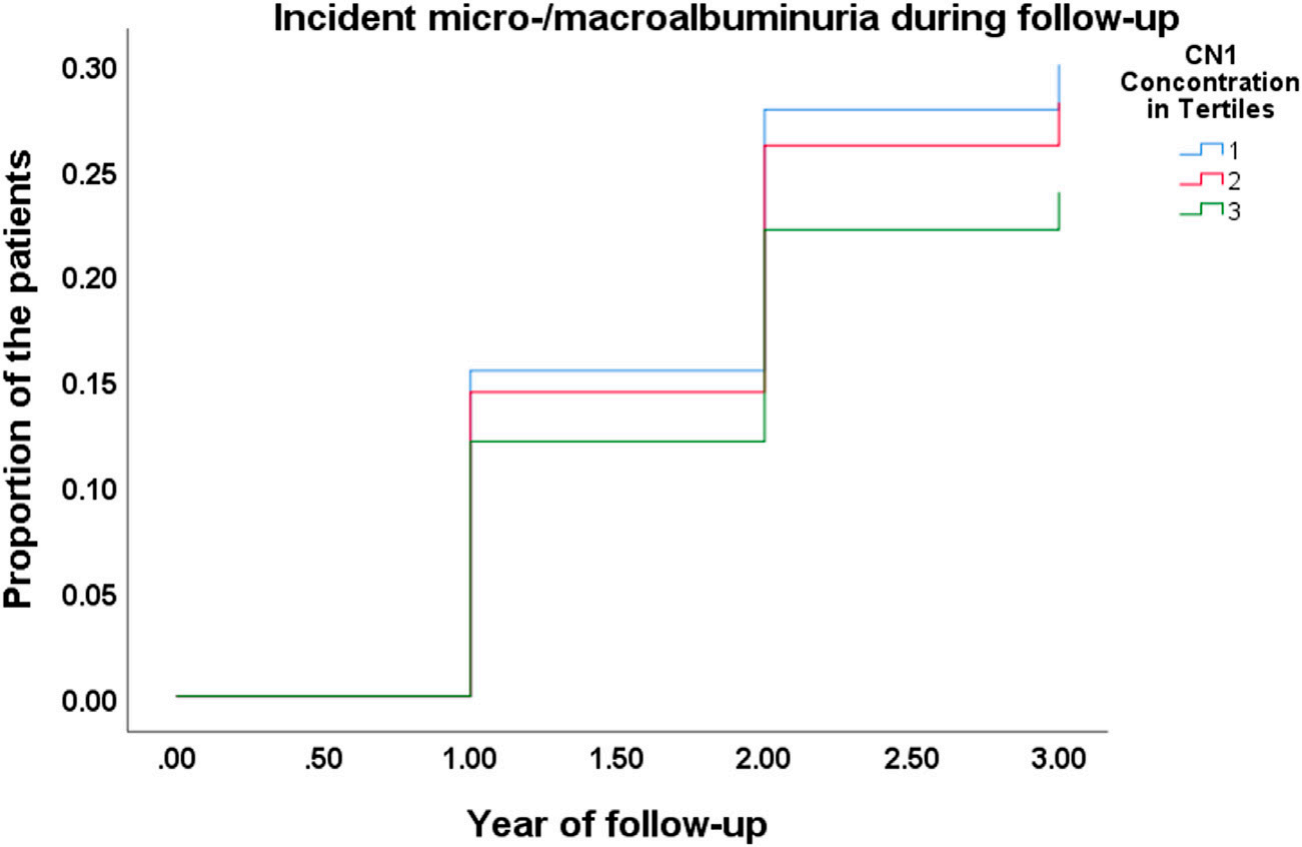
Kaplan-Meier curve for incident of micro-/macroalbuminuria during the follow-up in different CN1 tertiles.

A multivariable binary logistic regression analysis showed a significant association of CN1 concentration at baseline with the development of albuminuria during the follow-up (Χ^2^ for the model 12) = 21.249, *p* = 0.047, as shown in Figure 2). The results showed that for every unit in-crease in CN1 concentration the odds ratio for developing albuminuria was 0.673 (95% CI: 0.488–0.927, *p* = 0.015).

### Carnosinase 1 and eGFR Slope

We compared the serum CN1 concentration and activity levels in patients with an annual eGFR decline > 3 ml/min/1.73 m^2^ with those patients who had stable or moderately declining eGFR (GFR between −3 and +3 ml/min/1.73 m^2^ per anno). CN1 activity at baseline was significantly higher in patients with an annual eGFR decline of more than 3 ml/min/1.73 m^2^, U (N: 277 vs. 302) = 36,567, z = −2.616, *p* = 0.009, while CN1 concentration showed a similar trend (*p* = 0.054). After adjustment for age and gender, CN1 activity remained significantly higher in patients with a higher eGFR decline (*p* = 0.039), as shown in Figure 1B.

## Discussion

Previous studies have shown a genetic association between carnosinase-1 (CN1) and diabetic kidney disease (DKD) and demonstrated that patients with diabetes and certain CN1 genetic variants are more likely to develop DKD. However, serum CN1 concentration or activity had so far not been assessed as a diagnostic or prognostic parameter in a prospective cohort. In this work, we showed that CN1 concentration at baseline was significantly linked to renal function decline and onset of albuminuria during the follow-up period whereas CN1 activity was associated with eGFR slope.

In the current cohort, there was a significant but moderate association between serum CN1 concentration and activity. This moderate correlation may have resulted from possible posttranslational modifications differing between individuals that can influence the catalytic activity. As such, N-glycosylation has a strong impact on CN1 secretion and enzymatic activity (Riedl et al., 2010). Besides, CN1 is present both as a monomer and dimer in serum with different activity levels. Finally, other serum proteases cannot be excluded, as they may also contribute to carnosine degradation which was measured in our activity assay. All these points might explain the relatively moderate association between CN1 concentration and activity.

There was a significant association between use of calcium antagonist and CN1 activity. The highest percentage of patients taking calcium antagonist were found in the middle tertile. Currently we do not have an explanation for this relationship. Further studies will be required to investigate this association. There was no association between other medications and CN1 concentration or activity.

Serum CN1 concentration is associated with early DKD stage because it is sensitive towards proteinuria and may be decreased due to loss *via* urine (Rodriguez-Niño et al., 2019a; Rodriguez-Niño et al., 2019b; Zhang et al., 2019). In concordance with previous clinical findings, we found a positive association between baseline serum CN1 concentration and onset of renal function impairment during the follow-up period. Apart from age, gender, baseline serum creatinine, and HbA1c, serum CN1 concentration was also a significant prognostic factor of renal function impairment and onset of albuminuria in our prospective cohort study.

We also assessed eGFR decline, which is more robust measure of loss of renal function than the baseline eGFR (Vistisen et al., 2019). Higher baseline CN1 activities were associated with loss of eGFR whereas there was no association between eGFR decline and CN1 concentration. This was in concordance with previous preclinical studies showing a deleterious effect of higher CN1 activities (Sauerhöfer et al., 2007; Qiu et al., 2020).

These results support the hypothesis that a high CN1 activity depletes carnosine and other histidine-containing dipeptide, which were shown to be renoprotective by scaveng-ing reactive oxidative species and prevent the formation of advanced glycation end products (AGEs) (Bellia et al., 2014; Peters et al., 2017). Carnosine supplementation in rodents and patients has shown positive effects on glycoxidative parameters (de Courten et al., 2016; Regazzoni et al., 2016; Albrecht et al., 2017; Elbarbary et al., 2018; Houjeghani et al., 2018; Karkabounas et al., 2018).

Furthermore, carnosine scavenges acrolein or methylglyoxal, known reactive carbonyl species causing renal damage, and renders them unharmful (Carini et al., 2003; Aldini et al., 2005; Regazzoni et al., 2016; Deng et al., 2018). Carnosine reduces cell apoptosis and protects cells from acrolein-induced DNA damage.

Since carnosine is taken up orally and its concentration greatly depends on the serum CN1 activity, serum CN1 inhibition may be a therapeutic possibility to increase both serum carnosine and renal carnosine (Qiu et al., 2019). In rodents, CN1 inhibition may increase its renal concentration up to 100 fold (Qiu et al., 2019).

Previously, we have measured serum CN1 concentration and activity in a smaller cohort with 282 patients with T2DM of which 127 had DKD (Zhang et al., 2019). We found that serum CN1 differed between patients with DKD and those without. Within the patients with DKD, serum CN1 was associated with urinary carnosinase secretion (Rodriguez-Niño et al., 2019b). Interestingly, urinary CN1 secretion appears at an early stage of DKD, increases with the severity of DKD and was associated with serum CN1 (Rodriguez-Niño et al., 2019a). Thus, we hypothesized that serum carnosinase one might be a sensitive serum biomarker for early DKD. Compared to urine biomarkers, a sensitive serum biomarker for DKD might be more practical for clinical use, as it is easier to obtain and less dependent on circadian rhythm and volume status.

A major limitation of our study is the length of the observation period. Due to short duration of our study, with a mean follow-up period of 1.2 years, the number of patients developing renal impairment defined as eGFR below 60 ml/min/1.73 m^2^ (9.85%) and micro- or macroalbuminuria (15.78%) was relatively small. Likewise, we could not assess the influence of CN1 on doubling of serum creatinine or progression to end-stage renal disease. Further studies with longer follow-up period and longitudinal data on serum CN1 are desirable and could more robustly determine the prognostic value of serum CN1 for DKD progression. The cohort represents the general type 2 diabetes population in the Netherlands and may be extrapolated to other Caucasian populations in developed countries.

In summary, we measured CN1 activity and concentration in a large and well described T2D cohort (*n* = 970) and could detect significant associations between CN1 and renal function decline. This study gave the first evidence and more studies with more time points are desirable for further understanding.

## Data Availability

The raw data supporting the conclusions of this article will be made available by the authors, without undue reservation.
