# Supplementary material for: Association Between Serum Carnosinase Concentration and Activity and Renal Function Impairment in a Type-2 Diabetes Cohort
Source: Front Pharmacol. 2022 Jul 8;13:899057. doi: 10.3389/fphar.2022.899057 (PMC9304884; doi:10.3389/fphar.2022.899057)
Supplement: Supplementary file 1 [file DataSheet1.PDF]

# Supplemental Data to

## Association between serum carnosinase concentration and activity and renal function impairment in a type-2 diabetes cohort

**Jiedong Qiu [1], Benito A. Yard [2], Bernhard K. Krämer [3], Harry van Goor [4], Peter van Dijk [5,*] and Aimo Kannt [6,*]**

[1] 5th Medical Department, University Hospital Mannheim, Heidelberg University, D-68167 Mannheim, Germany; Department of Pathology and Medical Biology, University Medical Centre Groningen and University of Groningen, NL-9713 GZ Groningen, the Netherlands; jiedong.qiu@medma.uni-heidelberg.de

[2] 5th Medical Department, University Hospital Mannheim, Heidelberg University, D-68167 Mannheim, Germany; benito.yard@medma.uni-heidelberg.de

[3] 5th Medical Department, University Hospital Mannheim, Heidelberg University, D-68167 Mannheim, Germany; bernhard.kraemer@medma.uni-heidelberg.de

[4] Department of Pathology and Medical Biology, University Medical Centre Groningen and University of Groningen, NL-9713 GZ Groningen, the Netherlands;

[5] Department of Internal Medicine, University Medical Centre Groningen and University of Groningen, NL-9713 GZ Groningen, the Netherlands; Isala Diabetes Centre, NL-8025 AB Zwolle, the Netherlands; p.r.van.dijk@umcg.nl

[6] Institute of Experimental Pharmacology, Medical Faculty Mannheim, Heidelberg University, D-68167 Mannheim, Germany; Fraunhofer Institute for Translational Medicine and Pharmacology, D-60596 Frankfurt, Germany; aimo.kannt@itmp.fraunhofer.de

* Correspondence: Peter van Dijk (p.r.van.dijk@umcg.nl); Aimo Kannt (aimo.kannt@itmp.fraunhofer.de)

**Flow-chart of the cohort**

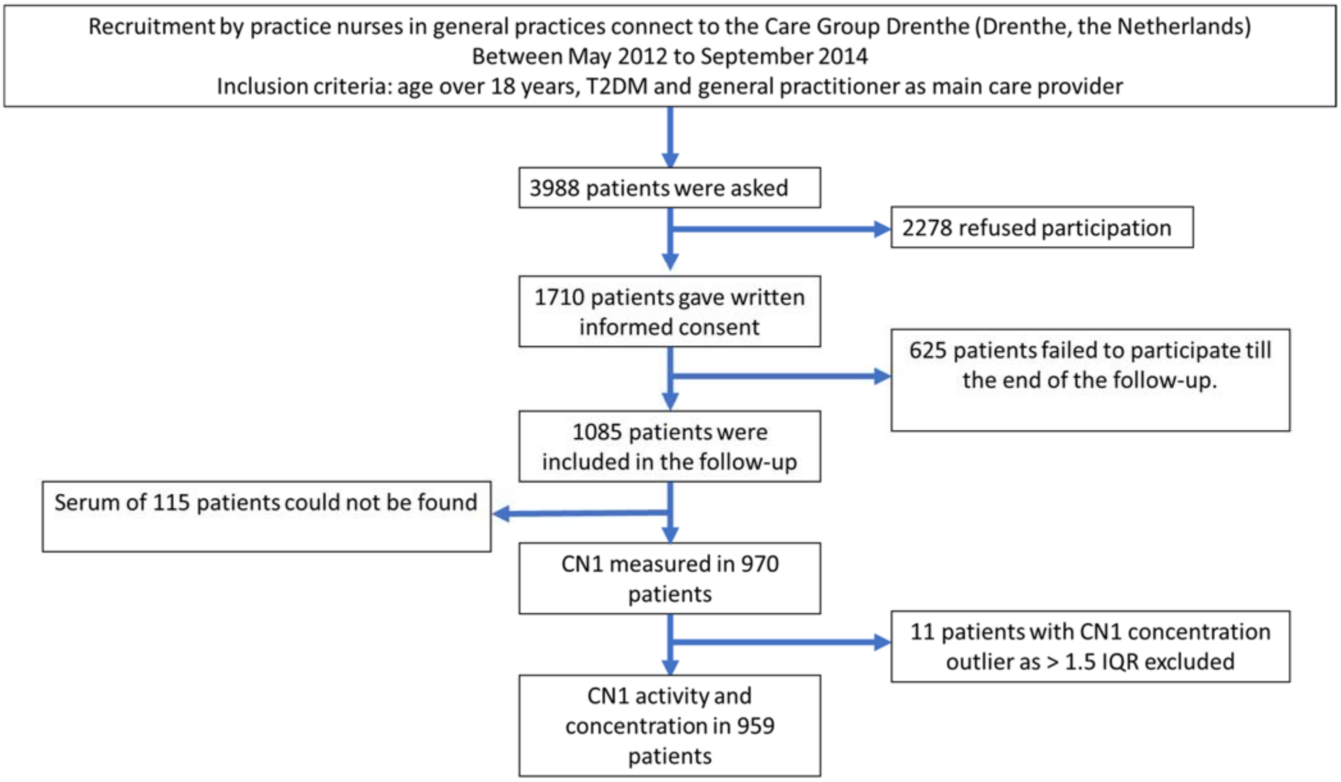

Supplemental Figure 1

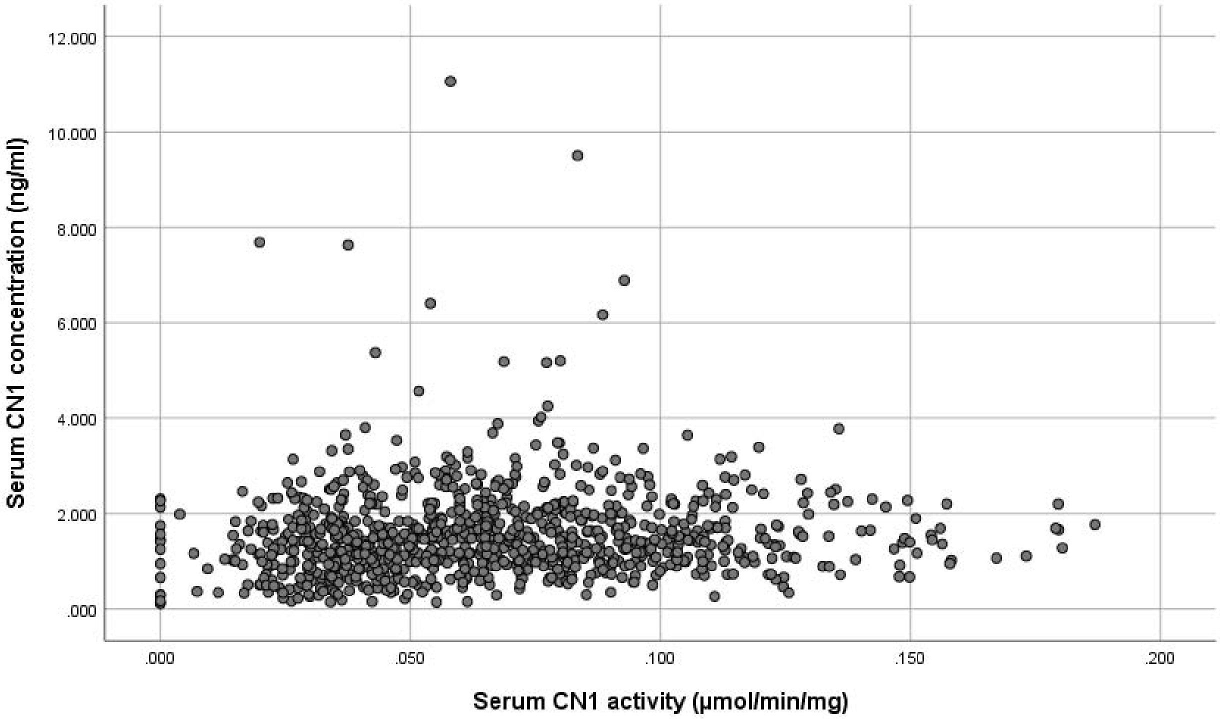


*Supplemental Figure 1. Scatter plot of the CN1 concentration and activity levels.*

**Supplementary Table 1.** Follow-up data at year 1 and CN1 concentration tertiles from baseline

| | CN1 concentration tertiles[b,c] | | | | | |
|---|---|---|---|---|---|---|
| | descriptives[a] | | tertile 1 (n = 320) | tertile 2 (n = 319) | tertile 3 (n = 320) | *p* - value |
| CN1 concentration [ng/ml] | 1.41 | [0.91] | 0.80 [0.39] | 1.41 [0.30] | 2.20 [0.72] | --- |
| Age [years] | 67.8 | [13.6] | 67.8 [15.0] | 68.1 [12.4] | 66.5 [14.6] | .457 |
| **Gender [% male]** | **54.9** | **% male** | **61.8%** | **54.9%** | **47.9%** | **.002** |
| HbA1c [mmol/mol] | 49 | [10] | 48 [10] | 49 [9] | 49 [9] | .057 |
| total cholesterol [mmol/l] | 4.3 | [1.2] | 4.3 [1.2] | 4.3 [1.25] | 4.3 [1.2] | .587 |
| HDL cholesterol [mmol/l] | 1.2 | [0.5] | 1.2 [0.4] | 1.2 [0.5] | 1.2 [0.4] | .760 |
| LDL cholesterol [mmol/l] | 2.3 | [1.0] | 2.3 [1.0] | 2.3 [1.1] | 2.4 [1.0] | .258 |
| triglycerides [mmol/l] | 1.5 | [1.0] | 1.5 [0.9] | 1.4 [1.1] | 1.5 [0.9] | .633 |
| SBP [mmHg] | 135 | [18] | 135 [20] | 135 [18] | 136 [17] | .616 |
| DBP [mmHg] | 78 | [12] | 78 [11] | 78 [12] | 80 [13] | .161 |
| **serum creatinine [µmol/l]** | **80** | **[24]** | **83 [24]** | **81 [22]** | **77 [23]** | **.001** |
| **eGFR MDRD [ml/min/1.73m²]** | **71** | **[23]** | **69 [22]** | **73 [23]** | **72 [23]** | **.046** |
| Urinary ACR [mg/mmol] | 0.8 | [1.1] | 0.9 [1.1] | 0.7 [1.1] | 0.8 [1.1] | .645 |

[a] descriptives as median [*IQR*] or frequency [%]

[b] tertiles as median [*IQR*] or frequency [%]

Abbreviations: CN1 carnosinase 1, BMI body mass index, HbA1c glycated hemoglobin, HDL high density lipoprotein, LDL low density lipoprotein, SBP systolic blood pressure, DBP diastolic blood pressure, eGFR estimated glomerular fitration rate, ACR albumin creatinine ratio

No data for BMI, diabetes duration and use of medications at Year 1

**Supplementary Table 2.** Follow-up data at year 1 and CN1 activity tertiles from baseline

| | descriptives[a] | | CN1 activity tertiles[b,c] tertile 1 (n = 319) | tertile 2 (n = 320) | tertile 3 (n = 320) | *p* - value |
|---|---|---|---|---|---|---|
| CN1 activity [μmol/min/mg] | 0.06 | [0.05] | 0.03 [0.01] | 0.06 [0.01] | 0.10 [0.03] | --- |
| Age [years] | 67.8 | [13.6] | 68 [14.2] | 68.5 [15.6] | 66.5 [11.1] | .727 |
| Gender [% male] | 54.9 | % male | 55.5% | 55.7% | 53.4% | .808 |
| HbA1c [mmol/mol] | 49 | [10] | 48 [10] | 49 [9] | 48 [9] | .596 |
| **total cholesterol [mmol/l]** | **4.3** | **[1.2]** | **4.2 [1.2]** | **4.3 [1.2]** | **4.4 [1.2]** | **.048** |
| HDL cholesterol [mmol/l] | 1.2 | [0.5] | 1.2 [0.5] | 1.2 [0.4] | 1.2 [0.5] | .868 |
| LDL cholesterol [mmol/l] | 2.3 | [1.0] | 2.2 [1.0] | 2.3 [0.9] | 2.4 [0.9] | .180 |
| **triglycerides [mmol/l]** | **1.5** | **[1.0]** | **1.4 [0.9]** | **1.5 [1.0]** | **1.6 [1.1]** | **.047** |
| SBP [mmHg] | 135 | [18] | 125 [22] | 135 [17] | 135 [14] | .908 |
| DBP [mmHg] | 78 | [12] | 78 [12] | 79 [12] | 78 [10] | .364 |
| serum creatinine [μmol/l] | **80** | **[24]** | 81 [23] | 81 [22] | 77 [26] | .142 |
| **eGFR MDRD [ml/min/1.73m²]** | **71** | **[23]** | **67 [20]** | **72 [23]** | **75 [27]** | **.000** |
| Urinary ACR [mg/mmol] | 0.8 | [1.1] | 0.7 [1.1] | 0.9 [1.1] | 0.9 [1.2] | .981 |

[a] descriptives as median [*IQR*] or frequency [%]

[b] tertiles as median [*IQR*] or frequency [%]

Abbreviations: CN1 carnosinase 1, BMI body mass index, HbA1c glycated hemoglobin, HDL high density lipoprotein, LDL low density lipoprotein, SBP systolic blood pressure, DBP diastolic blood pressure, eGFR estimated glomerular fitration rate, ACR albumin creatinine ratio

No data for BMI, diabetes duration and use of medications at Year 1

**Supplementary Table 3.** Follow-up data at year 2 and CN1 concentration tertiles from baseline

| | | | **CN1 concentration tertiles[b,c]** | | | |
|---|---|---|---|---|---|---|
| | descriptives[a] | | tertile 1 (n = 320) | tertile 2 (n = 319) | tertile 3 (n = 320) | *p* - value |
| CN1 concentration [ng/ml] | 1.41 | [0.91] | 0.80 [0.39] | 1.41 [0.30] | 2.20 [0.72] | --- |
| **Gender [% male]** | **54.9** | **% male** | **61.8%** | **54.9%** | **47.9%** | **.002** |
| HbA1c [mmol/mol] | 50 | [10] | 49 [12] | 50 [9] | 50 [11] | .871 |
| **total cholesterol [mmol/l]** | **4.4** | **[1.3]** | **4.5 [1.2]** | **4.5 [1.3]** | **4.0 [1.2]** | **.034** |
| HDL cholesterol [mmol/l] | 1.3 | [0.5] | 1.2 [0.3] | 1.3 [0.6] | 1.2 [0.4] | .162 |
| LDL cholesterol [mmol/l] | 2.3 | [1.2] | 2.5 [1.2] | 2.4 [1.4] | 2.2 [0.8] | .145 |
| triglycerides [mmol/l] | 1.5 | [1.0] | 1.6 [0.9] | 1.5 [1.1] | 1.5 [0.9] | .323 |
| SBP [mmHg] | 134 | [18] | 136 [19] | 134 [20] | 130 [20] | .117 |
| **DBP [mmHg]** | **76** | **[10]** | **74 [11]** | **78 [12]** | **77 [12]** | **.045** |
| **serum creatinine [µmol/l]** | **79** | **[24]** | **80 [23]** | **81 [22]** | **75 [22]** | **.026** |
| **eGFR MDRD [ml/min/1.73m²]** | **67** | **[22]** | **63 [22]** | **66 [18]** | **75 [28]** | **.004** |
| Urinary ACR [mg/mmol] | 0.9 | [1.2] | 1.0 [1.5] | 1.0 [1.6] | 0.6 [0.9] | .128 |

[a] descriptives as median [*IQR*] or frequency [%]

[b] tertiles as median [*IQR*] or frequency [%]

Abbreviations: CN1 carnosinase 1, BMI body mass index, HbA1c glycated hemoglobin, HDL high density lipoprotein, LDL low density lipoprotein, SBP systolic blood pressure, DBP diastolic blood pressure, eGFR estimated glomerular fitration rate, ACR albumin creatinine ratio

No data for age, BMI, diabetes duration and use of medications at Year 2

**Supplementary Table 4.** Follow-up data at year 2 and CN1 activity tertiles from baseline

| | descriptives[a] | | CN1 activity tertiles[b,c] tertile 1 (n = 319) | tertile 2 (n = 320) | tertile 3 (n = 320) | *p* - value |
|---|---|---|---|---|---|---|
| CN1 activity [µmol/min/mg] | 0.06 | [0.05] | 0.03 [0.01] | 0.06 [0.01] | 0.10 [0.03] | --- |
| Gender [% male] | 54.9 | % male | 55.5% | 55.7% | 53.4% | .808 |
| HbA1c [mmol/mol] | 50 | [10] | 50 [11] | 50 [11] | 51 [10] | .920 |
| total cholesterol [mmol/l] | 4.4 | [1.3] | 4.4 [1.2] | 4.3 [1.4] | 4.3 [1.1] | .618 |
| HDL cholesterol [mmol/l] | 1.3 | [0.5] | 1.2 [0.4] | 1.2 [0.5] | 1.4 [0.5] | .061 |
| LDL cholesterol [mmol/l] | 2.3 | [1.2] | 2.5 [1.2] | 2.3 [1.1] | 2.4 [0.9] | .527 |
| triglycerides [mmol/l] | 1.5 | [1.0] | 1.6 [1.1] | 1.5 [1.1] | 1.5 [1.1] | .812 |
| SBP [mmHg] | 134 | [18] | 134 [21] | 136 [18] | 130 [17] | .841 |
| DBP [mmHg] | 76 | [10] | 74 [10] | 76 [10] | 76 [10] | .862 |
| serum creatinine [µmol/l] | 79 | [24] | 79 [23] | 83 [30] | 77 [17] | .84 |
| eGFR MDRD [ml/min/1.73m²] | 67 | [22] | 64 [22] | 66 [19] | 70 [26] | .209 |
| Urinary ACR [mg/mmol] | 0.9 | [1.2] | 0.8 [1.0] | 1.0 [1.2] | 1.0 [1.6] | .974 |

[a] descriptives as median [*IQR*] or frequency [%]

[b] tertiles as median [*IQR*] or frequency [%]

Abbreviations: CN1 carnosinase 1, BMI body mass index, HbA1c glycated hemoglobin, HDL high density lipoprotein, LDL low density lipoprotein, SBP systolic blood pressure, DBP diastolic blood pressure, eGFR estimated glomerular fitration rate, ACR albumin creatinine ratio

No data for age, BMI, diabetes duration and use of medications at Year 2

**Supplementary Table 5.** Patient characteristics during the follow-up

| | baseline | Year 1 | Year 2 | *p* - value |
|---|---|---|---|---|
| HbA1c [mmol/mol] | 49 [10] | 49 [10] | 50 [10] | .363 |
| total cholesterol [mmol/l] | 4.3 [1.2] | 4.3 [1.2] | 4.4 [1.3] | .521 |
| HDL cholesterol [mmol/l] | 1.2 [0.5] | 1.2 [0.5] | 1.3 [0.5] | .613 |
| LDL cholesterol [mmol/l] | 2.3 [1.0] | 2.3 [1.0] | 2.3 [1.2] | .499 |
| triglycerides [mmol/l] | 1.5 [1.0] | 1.5 [1.0] | 1.5 [1.0] | .061 |
| SBP [mmHg] | 135 [19] | 135 [18] | 134 [18] | .024 |
| DBP [mmHg] | 80 [12] | 78 [12] | 76 [10] | .082 |
| **serum creatinine [µmol/l]** | **78 [22.25]** | **80 [24]** | **79 [24]** | **.038** |
| **eGFR MDRD [ml/min/1.73m$^2$]** | **73 [25]** | **71 [23]** | **67 [22]** | **.000** |
| **Urinary ACR [mg/mmol]** | **0.9 [1.1]** | **0.8 [1.1]** | **0.9 [1.2]** | **.012** |

[a] descriptives as median [*IQR*] or frequency [%]

Abbreviations: CN1 carnosinase 1, HbA1c glycated hemoglobin, HDL high density lipoprotein, LDL low density lipoprotein, SBP systolic blood pressure, DBP diastolic blood pressure, eGFR estimated glomerular fitration rate, ACR albumin creatinine ratio
